# COVID-19 Proned Ventilation and Its Possible Association With Foot Drop: A Case Series

**DOI:** 10.7759/cureus.14374

**Published:** 2021-04-08

**Authors:** Lawrence G Chang, Safwan Zar, Benjamin Seidel, Anupama Kurra, Andrew Gitkind

**Affiliations:** 1 Sports Medicine, Montefiore Medical Center, Bronx, USA; 2 Physical Medicine and Rehabilitation, Burke Rehabilitation Hospital, White Plains, USA; 3 Physical Medicine and Rehabilitation/Brain Injury Medicine, Burke Rehabilitation Hospital, White Plains, USA; 4 Physical Medicine and Rehabilitation, Montefiore Medical Center, Bronx, USA

**Keywords:** prone positioning, foot drop, compression neuropathy, electromyography, nerve conduction study (ncs), covid-19

## Abstract

The novel severe acute respiratory syndrome coronavirus 2 (SARS-CoV-2) virus causing the coronavirus disease 2019 (COVID-19) pandemic is known to lead to the complicated sequelae of severe acute respiratory distress syndrome. Proning has been used as an adjunctive treatment to improve oxygenation in both ventilated and non-ventilated patients. Although patients respond well to this strategy, complications from this arise as well. It is hypothesized that COVID-19 intensive care unit (ICU) proned ventilation is associated with new cases of foot drops or compressive unilateral ankle dorsiflexion weakness during the early 2020 COVID-19 pandemic. Five patients presented to an acute rehabilitation facility with unilateral ankle dorsiflexion weakness after ICU proned ventilation during the COVID-19 pandemic. Three patients were found to have primarily subacute left sensory-motor dysmyelinating common peroneal neuropathies located around the fibular head. Two patients were found to have primarily subacute sensory-motor dysmyelinating right-sided common peroneal neuropathies above the fibular head and distal to biceps femoris muscle. Compressive unilateral common peroneal neuropathies during the pandemic are possibly related to the impromptu, unconventional, and unfamiliar use of proned ventilation.

## Introduction

The coronavirus disease (COVID-19) virus can cause a spectrum of sequelae, the most serious being fatal acute respiratory distress syndrome (ARDS) and multi-organ failure in patients worldwide. The prevalence of ARDS in COVID-19 patients during early 2020 was about 17% [1]. One essential strategy in combating COVID-19-related ARDS includes proned ventilation, a non-conventional infrequently used intensive care unit (ICU) technique to improve oxygenation and reduce ventilatory lung injury [2]. Proning is a usually safe adjuvant treatment in both intubated and non-intubated patients to improve oxygenation and decrease mortality in ARDS [2-6].

Many hospitals have developed their own variation of the proning protocol. One of the commonly used proning methods is the “Cornish Pasty” technique [7]. The “Cornish Pasty” technique is described in the following order: (1) team plans procedure, (2) sheets are placed over and under the patient, (3) pillows are placed under the chest and pelvis, (4) pressure areas are checked to be protected, (5) lines are checked and made safe/secured, (6) team wraps the patient in sheets, (7) the patient is turned in a lateral recumbent position, (8) lines and vitals are checked and secured safely, and (9) the patient is ultimately proned. After being proned, the patient is maintained in the “swimmer’s position” or “swimmer’s crawl” where the head is facing in one direction with the ipsilateral arm raised up and the ipsilateral hip and knee flexed [7-11]. The contralateral arm and leg are placed extended alongside the patient’s body [7,8]. A pillow is placed on the shoulder, chest, and hip ipsilaterally to where the patient is facing [7,8]. Alternatively, the patient can be placed in a “modified swimmer’s” position where the arms are bilaterally placed on the sides of the patient [10]. Pressure areas are reassessed with a cushion placed on face, palms, patella, and pre-tibial regions [7,8]. The patient is then placed in reverse Trendelenburg position [7], and the ankles are placed neutrally with the feet offloaded [9-11].

Proning is maintained with body positions alternated on an average of every 2-4 hours for 16-18 hours prone in either the swimmer’s position or other positions where the limbs are adjusted to avoid common pressure points [7-11]. Ghelichkhani and Esmaeili in 2020 recommended the need to change patient positioning and switch sides every two hours with multiple healthcare providers to prevent complications [12]. The optimal methodology, duration, and timing of proning in ventilated COVID-19 patients during the pandemic is currently unknown.

Given the assumed need for long duration of this position to maximize benefits, the side effects of this maneuver may have been overlooked, resulting in complications such as brachial plexus injuries, ischemic optic neuropathy, decubitus ulcers, tube dislodgements, transient desaturations, and compressive neuropathies [4,5,11-16].

Compressive neuropathies associated with mechanical ventilation in prone positioning are rarely cited in the literature. Some risk factors for neuropathies include severe weight loss, diabetes mellitus, habitual leg crossing, compressive masses, and fibrous bands [16]. Electrodiagnostic studies are used to help diagnose, localize, determine severity, and monitor recovery after a nerve injury has been identified [17].

Although patients with COVID-19-related ARDS respond well to proning, complications have been identified in the literature, and are not limited to dermatological, cardiopulmonary, and neuromusculoskeletal conditions [18]. It is hypothesized that new cases of compressive unilateral ankle dorsiflexion weakness may be associated with a course of ICU proned ventilation during the early 2020 COVID-19 pandemic.

## Case presentation

Cases were obtained from the inpatient rehabilitation facility and acute hospital electronic medical records. Cases included in the study had the following criteria: (1) a history of being COVID-19 positive, (2) a history of ICU proning, (3) unilateral ankle dorsiflexion weakness, (4) admission to an acute inpatient rehabilitation facility from March to June 2020, (5) recent discharge from an acute care hospital from March to June 2020, and (6) electromyography (EMG) and nerve conduction studies (NCS) conducted to elucidate the etiology of unilateral ankle dorsiflexion weakness. The exclusion criteria for the cases were no EMG/NCS conducted, premorbid unilateral/bilateral limb or ankle dorsiflexion weakness, prior neuropathy, severe weight loss, prior nutritional deficiencies, compressive masses, or cancer metastasis. Institutional Review Board approval was not necessary given the fact that all care was performed according to the standard medical practice and that these trends were noted retrospectively. Five patients were found to satisfy the above inclusion criteria. See Table 1 for patient demographics.

**Table 1:** Patient demographics. EMG: electromyography; NCS: nerve conduction studies

Age	Gender	COVID-19 history	Proned	Medical co-morbidities	Foot drop	Side	EMG/NCS conducted
63	Male	Yes	Yes	Hypertension	Yes	Left	Yes
68	Male	Yes	Yes	Hypertension, hyperlipidemia, hypothyroidism	Yes	Left	Yes
55	Female	Yes	Yes	Hypertension, chronic obstructive pulmonary disease	Yes	Left	Yes
55	Male	Yes	Yes	Human immunodeficiency virus, hepatitis C virus, chronic obstructive pulmonary disease	Yes	Right	Yes
30	Male	Yes	Yes	Obesity, active smoker	Yes	Right	Yes

**Figure 1:**
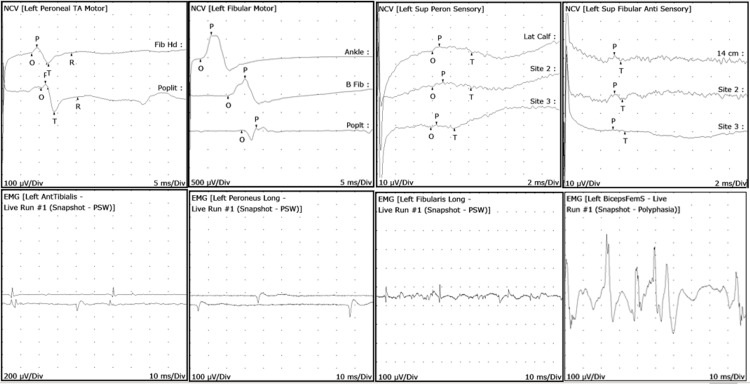
EMG/NCS left lower extremity representative waveforms. EMG: electromyography; NCS: nerve conduction study Top left two figures (NCS): Left peroneal/fibular motor nerve conduction block noted in popliteal regions indicating compressive neuropathy Top right two figures (NCS): Left superficial peroneal/fibular sensory nerve conduction is poor and seen with inconsistent responses Bottom left two figures (EMG): Left anterior tibialis muscle with positive sharp waves (PSWs) and fibrillations (Fibs). Left peroneus longus muscle with PSWs. These indicate acute denervation of the muscles Bottom right two figures (EMG): Left peroneus/fibularis longus muscle with PSWs which indicates acute muscle denervation. Biceps femoris muscle with polyphasic potentials which indicate subacute denervation of muscle

Three patients on EMG/NCS had primarily subacute left sensory-motor, primarily dysmyelinating common peroneal neuropathies. Two patients had right-sided primarily subacute sensory-motor dysmyelinating common peroneal neuropathies. Three of the left-sided injuries were localized at or above the common peroneal nerve with conduction blocks at the superficial peroneal nerve. Two patients were found to have primarily sensory-motor dysmyelinating right-sided common peroneal neuropathy above the fibular head and distal to biceps femoris muscle. One patient had sural nerve involvement. Needle examination revealed abnormal potentials at peroneal innervated muscles. None of the patients revealed lumbar radiculopathy or plexopathy. See Figure 1 for representative left lower extremity EMG/NCS waveforms. All patients had improvements of unilateral ankle dorsiflexion weakness after conventional rehabilitation.

## Discussion

It is possible that all patients with unilateral ankle dorsiflexion weakness developed them during the early 2020 COVID-19 pandemic with proned ventilation. The overwhelming hospital surge created a widespread lack of healthcare providers to turn patients in a timely manner. This quick impromptu implementation of an uncommonly and unfamiliarly used proned protocol possibly resulted in pressure-related injuries. Healthcare providers’ fears of entering the room frequently to change positions due to severe lack of personal protective equipment and uncertainty of viral infectivity while in the room may have been other factors contributing to poor adherence to the proning protocol, and thus, prolonged compressive neuropathy. The EMG/NCS studies and the observation of clinical improvement of patients after conventional rehabilitation support the possibility that the etiology was unilateral compressive neuropathy of the common peroneal nerve.

It is still suspected that laterality may be related to positioning, which brings to light that the “swimmer’s position” should be modified to prevent compression neuropathy. Other metabolic and medical factors are not entirely excluded. COVID-19 pathological side effects may play some unknown role in exacerbating conditions for unilateral ankle dorsiflexion weakness. Weight loss may have also played a role but occurs via a different pathological mechanism. Studies have shown that weight loss-associated foot drops are usually seen with habitual cross-legging, its predilection for both unilateral and bilateral nerve compression, and prior history of weight loss of greater than 25 pounds usually due to dieting, bariatric surgery, and anorexia [19,20].

## Conclusions

In conclusion, the cases presented here on the impromptu, unconventional, and unfamiliar use of proned ventilation during the early 2020 COVID-19 pandemic are possibly associated with new cases of compressive unilateral common peroneal neuropathy. In the future, larger-scale studies need to be conducted to validate this association. Improving the detection and reduction of compressive neuropathies may start with further investigation of the safety of the proning protocol. Rehabilitation from any illness is often a long process, and similarly, compressive neuropathies can be debilitating, and it may take up to a year to achieve maximal healing. While proning should continue to be utilized as dictated by the current literature, changing patient positioning at shorter intervals should continually be emphasized along with the early inclusion of physiatrists to better evaluate the need for timely mobilization. Individualized institutional guidelines should be developed and discussed with all involved.
